# Novel pretransplant desensitization strategies in heart transplantation

**DOI:** 10.1016/j.jhlto.2025.100242

**Published:** 2025-03-06

**Authors:** Guillaume Coutance, Anita S. Chong, Marlena V. Habal

**Affiliations:** a Department of Cardiac and Thoracic Surgery, Cardiology Institute, Pitié Salpêtrière Hospital, Assistance Publique-Hôpitaux de Paris (AP-HP), Sorbonne University Medical School, Paris, France; b Paris Translational Research Centre for Organ Transplantation, INSERM, UMR-S970, University Paris Cité, Paris, France; c Department of Surgery, University of Chicago, Chicago, Illinois; d Division of Cardiology, Department of Medicine, New York University, Grossman School of Medicine, New York, New York

**Keywords:** heart transplantation, desensitization, antibody-mediated rejection, immunosuppression, immunology

## Abstract

Allosensitization remains a major barrier in thoracic organ transplantation, limiting access to transplantation and increasing waitlist mortality and post-transplant morbidity. Desensitization protocols aimed at improving access to transplantation and mitigating the risk of early post-transplant rejection have been developed, but current strategies have limited efficacy, and new strategies are needed. After a synthetic description of the basics of alloimmune responses leading to the production of donor-specific antibodies, the potential of novel desensitization strategies, including anti-CD38 therapies, costimulation blockade, and interleukin-6 inhibition as pretransplant desensitization therapies, are discussed in detail, including the rationale for their use, results of preclinical and clinical studies, and potential practical clinical application. Complementary novel pharmacologic (individualization therapies, combination desensitization therapies, additional perioperative antibody-risk mitigation strategies) and nonpharmacologic strategies (individual risk stratification and combination of immunologic assays) are also presented. Finally, potential next-generation therapies (bispecific T-cell engager and chimeric antigen receptor T cells) and clinical outcomes of interest are briefly discussed. Overall, this review aims to provide recent data on this constantly evolving field, while keeping in mind the clinical applicability and providing practical aspects of the use of novel pretransplant desensitization therapies.

## Background

Allosensitization presents a major obstacle to thoracic organ transplantation with 2 important issues: (1) on the waiting list, the higher the sensitization, the lower the access to transplantation and the higher the mortality,^1^ and (2) early after transplantation, crossing the human leukocyte antigen (HLA) barriers by transplanting against donor-specific antibodies (DSA) have been associated with increased mortality and morbidity, including early antibody-mediated rejection (AMR) and cardiac allograft vasculopathy (CAV).^2^, ^3^ Desensitization protocols aimed at improving access to transplantation and mitigating the risk of early post-transplant AMR have been developed.^4^, ^5^ Most transplant centers have a dedicated high-immunological risk program, one third of them considering desensitization for calculated panel reactive antibody (cPRA) levels of 50% to 80%, with another third for cPRA above 80%. Current common desensitization approaches include plasmapheresis/immunoadsorption, intravenous immunoglobulins (IVIg), rituximab, and bortezomib.^5^ The complexity and redundant immune pathways may explain the limited efficacy of conventional desensitization therapies, and understanding these pathophysiological mechanisms is crucial. While new immunosuppressive drugs for solid-organ transplants are lacking, recent progress in treating hematologic cancers and autoimmune diseases has introduced new strategies for managing allosensitization, as these conditions share similar mechanisms including T-cell and B-cell differentiation and complement activation. Understanding these mechanisms is crucial, and this review will discuss the rationale for clinically relevant strategies as well as emerging strategies in heart transplant care.

## Alloimmune response to the exposure of foreign HLA antibodies

**Figure 1:**
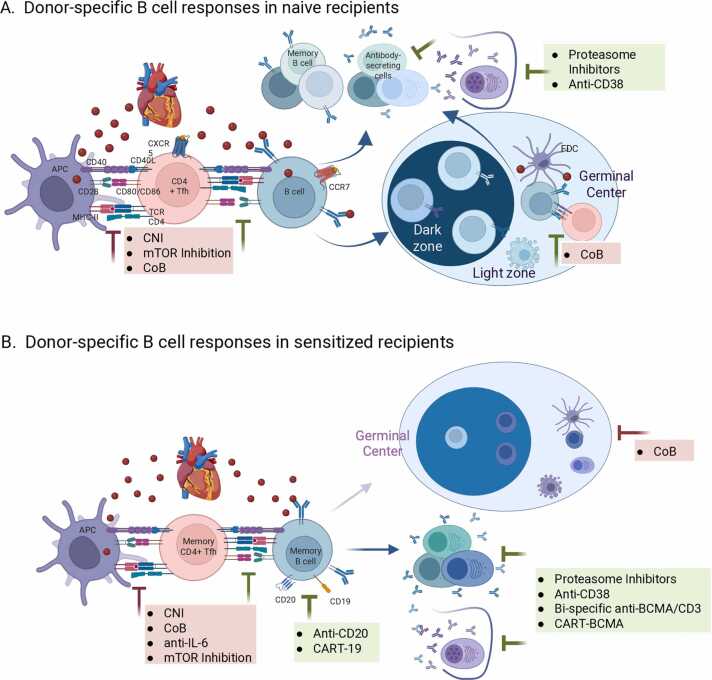
Immune response to the exposure to foreign anti-HLA antibodies. Depiction of B cell differentiation upon encounter with donor antigens in (A) naive or (B) sensitized recipients. The germinal center reaction plays an important role in generating affinity-matured plasmablasts and plasma cells in naive recipients, while the extrafollicular response dominates in sensitized recipients. The rationale for combining therapies to selectively inhibit T follicular helper differentiation and to selectively inhibit B-cell activation, germinal center B-cell responses or depleting plasmablasts or plasma cells with the indicated immunosuppressive agents. Red inhibition arrow: T-cell-directed immunosuppression. Green inhibition arrow: B-cell, plasmablast, or plasma cell-directed immunosuppression. APC, antigen-presenting cells; CoB, costimulation blockade; FDC, follicular dendritic cells; TCR, T-cell receptor; Tfh, T follicular helper cells.

Upon allograft transplant in naive recipients, B-cells located within the follicles of secondary lymphoid organs that encounter alloantigen downregulate the chemokine receptor CXCR5 and upregulate CCR7 to migrate to the T:B cell border (Figure 1).^6^, ^7^ There B-cells engage in cognate interactions with CD4^+^ T-cells that had been activated by alloantigen presented indirectly by recipient antigen-presenting cells (APCs) and had upregulated CXCR5. This encounter provides B-cells with survival signals through CD40 and IL-21 that together stimulates their differentiation into short-lived antibody-secreting plasmablasts, memory B-cells, or into cells that upregulate CXCR5 and return to the B-cell follicle to enter into germinal centers (GC) reactions. In GCs, B-cells undergo somatic hypermutation and proliferation in the dark zone, competing for help from GC T-follicular helper cells. Eventually, they differentiate as post-GC memory B-cells, plasmablasts, and long-lived plasma cells. T-cell help is necessary for donor-specific IgG production by pre- and post-GC B cells can be inhibited by conventional immunosuppression or costimulation blockade (CoB). However, once plasmablasts or plasma cells are formed, proteasome inhibitors or anti-CD38 antibodies are needed to deplete these cells and halt further DSA production.^8^, ^9^

In sensitized recipients, B-cell responses to allografts differ significantly. Even in the case of perioperative management of preformed DSA or in sensitized patients with undetectable DSA at the time of transplant, patients may still experience acute AMR due to memory T- and B- cells that resist calcineurin-inhibitors and antiproliferative drugs.^8^ Preclinical studies have shown that upon alloantigen re-exposure, alloreactive memory B-cells rapidly differentiate into plasmablasts in a predominantly GC-independent manner and that this recall response is prevented by CTLA-4Ig (Figure 1).^10^ At the same time, naive and lower-affinity memory B-cells may enter the GC during the recall response.

Historically, patients with high-titer DSA were excluded from receiving a transplant due to the risk of hyperacute rejection. Perioperative antibody-risk mitigation strategies can remove or neutralize DSA, but the effects are temporary without targeting DSA-producing cells. Long-lived plasma cells are responsible for protracted DSA production, so their depletion was initially attempted with proteasome inhibitors. However, plasma cell depletion was reported to be insufficient as DSA rebounded after treatment was stopped, and in fact induced humoral compensation characterized by increased B-cell and T-follicular helper responses in nonhuman primates.^11^, ^12^ These observations raised the possibility that donor-specific APCs were continuously generated to rapidly replace the cells being depleted. Since long-lived plasma cells generally do not proliferate significantly in response to antigens, the rationale is that new antibody-secreting cells are constantly being produced by differentiating memory B cells receiving T cell help and that this can be inhibited by CoB.

## Novel pretransplant pharmacologic desensitization strategies

**Figure 2:**
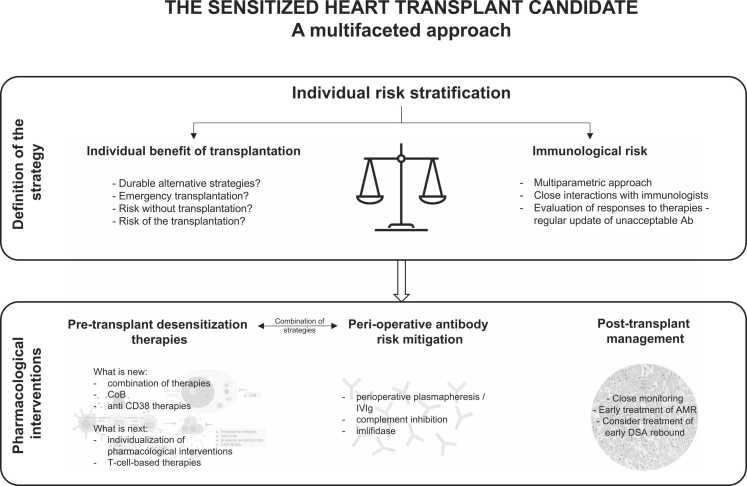
The sensitized heart transplant candidate. A multifaceted approach. Synthesis of the combination of strategies for the sensitized heart transplant candidates. AMR, antibody-mediated rejection; CoB, costimulation blockade; DSA, donor-specific antibodies; IVIg, intravenous immunoglobulins.

**Figure 3:**
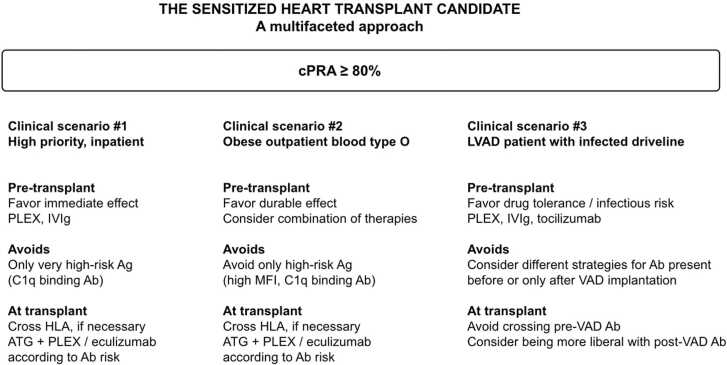
The sensitized heart transplant candidate: various clinical scenarios. Three clinical scenarios are discussed. Despite a similar degree of pretransplant sensitization, the proposed strategy differs greatly according to the degree of emergency of transplantation, the access to transplant, and the infectious risk. Clinical scenario #1: for a high-priority patient, an emergency procedure compatible with rapid transplantation is proposed. Crossing DSA at transplant should be considered as a possible standard procedure. Clinical scenario #2: for a patient with low access to transplantation, a procedure with a potential durable effect is proposed. Crossing DSA at transplant should be considered. Clinical scenario #3: for a patient with an infected durable mechanical circulatory support, the risk/benefit ratio of sensitization should be assessed individually. Due to recent data suggesting that post-LVAD de novo anti-HLA antibodies might drop rapidly after transplant, a more liberal approach for antibodies is present only after implantation. Ab, antibody; Ag, antigen; ATG, antithymocyte globulin; IVIg, intravenous immunoglobulins; MFI, mean fluorescence intensity; PLEX, plasma exchange; VAD, ventricular assist device.

The desensitization protocols that will be further discussed in this chapter are based on the off-label use of therapies that are Food and Drug Administration- or European Medicines Agency-approved in other clinical settings. Importantly, data in the field, including kidney transplantation, mostly relies on phase I/II open-label noncontrolled clinical studies. Novel therapies are often considered as add-on therapies for highly sensitized candidates nonresponsive to standard desensitization approaches, making it even more challenging to evaluate accurately the specific effect of the new molecule (Figure 2, Figure 3).

### Anti-CD38 therapies

Daratumumab and isatuximab are anti-CD38 monoclonal antibodies that target different epitopes of CD38, a glycoprotein present on the surface of numerous immune cells, including plasma cells, plasmablasts, NK cells, and a subset of potent immunosuppressive regulatory T cells.^13^ CD38 plays an important role in cell adhesion, signal transduction, and calcium signaling. Both drugs promote antibody-dependent cellular cytotoxicity, complement-dependent cytotoxicity, and cellular phagocytosis. Currently approved for treating multiple myeloma, these drugs are typically combined with other therapies, especially for patients who have undergone prior treatments.

In allosensitization, targeting plasma cells (antibody producers) and NK cells (key in AMR) could be advantageous (Table 1).^14^ A nonhuman primate study showed daratumumab reduced memory B and plasma cells, lowered DSA levels, and prolonged renal graft survival, though DSA reduction was not sustained. Early clinical use also showed temporary antibody reduction.^15^, ^16^, ^17^ A phase I/II study with isatuximab indicated adequate reduction in CD38+ cells and HLA-specific IgG memory B cells, although cPRA values only dropped to target levels in 39% of patients, but with sustained antibody decreases in most responders for 26 weeks.^17^ In phase I/II kidney transplant desensitization study (23 patients), daratumumab showed a good safety profile and transient anti-HLA antibody reductions, with under 40% achieving durable responses.^15^ Although transient, an early reduction in cPRA may create a valuable window for thoracic transplants regarding the shorter waiting time compared to kidney transplantation, with ongoing phase I/II preheart transplant studies.^18^, ^19^

**Table 1:** Novel Pretransplant Desensitization Therapies and Perioperative Antibody-Risk Mitigation Strategies: Summary of Main Clinical Studies

Drug	FDA-approved indications	Preclinical studies	Clinical studies: kidney transplantation	Clinical studies: heart transplantation
Anti-CD38 therapies (References: 17, 18, 19, 20, 21, 22) daratumumab, isatuximab	Multiple myeloma	− Reduced memory B-cells and plasma-cell populations − Reduced DSA levels − Prolonged renal allograft survival	− Transient anti-HLA antibody reductions − ∼40% responders − Good safety profile	− Case reports − Ongoing phase I/II studies
Costimulation blockade (References: 9, 11, 23, 24, 25, 26, 27) belatacept	Prophylaxis of organ rejection in adults kidney transplantation	− Reversal of established donor-specific B-cell responses − Disruption of germinal centers − Prevention of further B-cell activation. − On top of a proteasome-inhibitor: reduction of DSA and improved post-transplant survival	− Combination of therapies − Phase I/II studies − Good safety profile − Reduced anti-HLA Ab − Broad elimination of HLA antibodies not realized and long-term data needed	− Case series of 4 patients (cPRA >99%): belatacept + proteasome inhibitor = increased number of potential donors, transplants across C1q-binding DSA
Anti-IL-6 therapies (References: 28, 29, 30, 31, 32, 33) tocilizumab, clazakizumab	Tocilizumab: rheumatoid arthritis, giant cell arteritis, polyarticular and systemic juvenile idiopathic arthritis, and cytokine release syndrome Clazakizimab: no-FDA-approved indications	− Blocking IL-6 combined with costimulation blockade improves allograft tolerance − IL-6 receptor blockade with carfilzomib delayed DSA rebound post transplant	− Minimal impact of anti-IL-6 monotherapy − On top of other therapies for nonresponsive candidates: reduction in MFI, prevention of DSA rebound after transplant, increased relative frequencies of post-transplant T-reg and B-reg cells	− Experience mostly based on a post-transplant use of IL-6 therapies − Lack of data on the pretransplant use
Complement inhibition (References: 34, 35) eculizumab	− Paroxysmal nocturnal hemoglobinuria − Atypical hemolytic uremic syndrome − Generalized myasthenia gravis − Neuromyelitis optica spectrum disorder	− Effective blockade of complement hemolytic activity − Long-term cardiac allograft survival despite the persistence of DSA − Promotion of accommodation?	− Consistent decrease in the risk of early active AMR − Concerns regarding the high incidence of chronic lesions (chronic active AMR)	− Favorable outcomes − Compared to the standard of care group, significant decrease in the risk of AMR − No signals concerning chronic lesions (graft dysfunction, cardiac allograft vasculopathy)
Imlifidase (References: 36, 37)	− Desensitization of highly sensitized adult kidney transplant recipients		− Very-high risk transplantations − DSA rebound ++ − Frequent early active AMR responsive to treatment	− No experience reported (a case report in lung transplantation)

Ab, antibody; AMR, antibody-mediated rejection; cPRA, calculated panel reactive antibody; DSA, donor-specific antibody; FDA, Food and Drug Administration; MFI, mean fluorescence intensity.

Potential limits: daratumumab may not deplete all antibody-producing cells, especially long-lived plasma cells in bone marrow niches.^20^ These cells may continue producing alloantibodies, and immune reconstitution could lead to a rebound effect. The depletion of regulatory CD38+ immune cells may contribute to increase the risk of rejection.^21^

### Costimulation blockade

The limited effectiveness and tendency for rebound after plasma cell-depleting therapies underscore the need for synergistic treatments to better control B-cell differentiation. T-cell costimulation via CD28/B7 and CD40L/CD40 pathways is essential for T-dependent B-cell responses, including those directed against HLA antigens. Early studies showed that CD28/B7 blockade with CTLA4-Ig or belatacept prevented humoral sensitization, with a low incidence of de novo DSA.^22^ More recent research highlights the role of CoB in attenuating B-cell memory, with studies in mice demonstrating that CoB (anti-CD40 or CTLA4-Ig) can reverse established donor-specific B-cell responses, disrupt GCs, and prevent further B-cell activation.^8^, ^10^, ^38^ Nonhuman primate studies also showed that adding CoB to a proteasome-inhibitor-based regimen reduced DSA and improved post-transplant survival.^39^, ^40^ Treatment resulted in a reduction in plasma cells and T-follicular helper cells which may have contributed to the observed response. These findings position CoB as playing a distinct role in reversing established humoral alloimmunity.

In a proof-of-concept case series, belatacept and a proteasome inhibitor were used to desensitize 4 highly sensitized heart transplant candidates (cPRA >99%), increasing the number of potential donors and enabling transplants across C1q-binding DSA.^41^ These protocols used standard doses of bortezomib 1.3 mg/m^2^ or carfilzomib 20 to 36 mg/m^2^ along with belatacept at doses recommended for kidney transplantation. Collectively these studies highlight several key considerations. First, CoB alone cannot fully reverse established responses once plasma cells have developed and thus belatacept should be used synergistically with plasma cell-targeted therapies.^8^, ^23^ Second, effective desensitization must be paired with tailored post-transplant strategies. In sensitized nonhuman primates, only when belatacept was continued post transplant, was survival meaningfully extended.^24^ In our protocols, desensitized transplant recipients remain on belatacept post transplant; at doses commensurate to those used in kidney transplantation, although the dosing strategy is tailored to the individual patient. Lastly, as belatacept was initially designed to block T-cell activation, its optimal dose and timing for desensitization and peritransplant use still need refinement (Table 1).

Potential limitations: As outlined above, CoB requires upfront use in combination with a plasma cell-depleting strategy. Current Food and Drug Administration-licensed CoB is limited to blocking the CD28-B7 pathway. However, attenuating CD40/CD40L interactions may be even more effective.^25^ Finally, the optimal CoB-inclusive regimen for presensitized individuals after transplant has not been defined.

### Interleukine-6 inhibition

Interleukine-6 (IL-6) is a central cytokine in regulating inflammation and the development, maturation, and activation of T cells, B cells, and plasma cells. Anti-IL-6 therapies may offer long-term control of B-memory and plasma cell DSA responses thus limiting graft injury.^26^ Tocilizumab is a humanized monoclonal antibody that targets and inhibits both soluble and membrane-bound forms of the IL-6 receptor. Clazakizumab is a humanized monoclonal antibody that inhibits IL-6 by specifically binding to the cytokine itself.

Preclinical studies show that IL-6 is elevated during allograft rejection, and blocking IL-6 combined with costimulation blockade improves allograft tolerance (Table 1). In sensitized primate models, IL-6 receptor blockade with carfilzomib delayed DSA rebound post transplant.^27^

In 13 kidney transplant candidates, tocilizumab as monotherapy minimally affected anti-HLA alloantibodies.^42^ In a single-center trial with 10 sensitized candidates unresponsive to IVIg and rituximab, tocilizumab combined with IVIg reduced DSA mean fluorescence intensity (MFI) at transplant and 1 year later.^43^ In a similar group, clazakizumab enabled transplantation in 18 of 20 patients without DSA rebound and increased the relative frequencies of post-transplant Treg and Breg cells.^44^ While initial studies suggested a potential favorable impact of IL-6 inhibition in the treatment of chronic active AMR after kidney transplantation, the first large-scale randomized trial was terminated early for futility (clazakizumab, IMAGE trial).^28^

In thoracic transplant cases, data on tocilizumab for desensitization or AMR treatment are limited. A series of 7 patients reported an average 31% reduction in dominant antibody levels. Tocilizumab has also been used in early postoperative management with other therapies, though its specific impact remains uncertain.^29^ A randomized clinical trial is assessing tocilizumab with standard immunosuppressive regimens post-HTx, focusing on dnDSA, rejection, and survival at 12 months.^30^

Beyond acute allograft rejection, IL-6 also plays a key role in the development of CAV.^31^, ^32^, ^33^ Considering that sensitized candidates transplanted across the HLA barrier may be at increased risk of CAV,^3^ IL-6 inhibition might be particularly relevant in this context. By shifting the immune profile toward a state of tolerance, these agents may thus prevent the long-term sequelae limiting graft survival, such as chronic rejection, particularly by enhancing the function of Tregs and increasing their intra-graft number, reducing Th17-mediated chronic inflammation, decreasing B-cell maturation and potentially DSA production, and dampening innate immune activation.^45^, ^46^, ^47^ However, the impact of IL-6 inhibition on CAV progression is currently under investigation (ALL IN trial).

Limitations: Clinical studies suggest that anti-IL-6 agents used as monotherapy may have limited effects since they are not depleting agents or robust inhibitors able to profoundly alter the alloimmune response.

## Novel complementary pharmacologic and nonpharmacologic strategies

While novel pharmacologic strategies are crucial in improving desensitization, the consideration of a broader picture and overall transplant journey is essential to guide therapies. In the same way that our post-transplant follow-up is gradually evolving toward individualization rather than a protocol-based approach, our pre- and peritransplant desensitization strategy should also evolve in this direction (Figure 2).

### Multimodal assessment of antibodies—Key role of immunologists

While isolated MFI values have, in numerous patients, technical and practical advantages when deciding to desensitize or to select the appropriate graft for a given patient, they should be combined with complementary tests in highly sensitized patients to provide a more comprehensive picture of the immunologic risk and the potential answer to perioperative antibody-risk mitigation strategies (dilution, C1q-binding, surrogate crossmatches).^48^, ^49^ A too stringent and conservative definition of unacceptable antibodies may dramatically limit access to transplantation and impact patient outcomes. The active participation of immunologists in pretransplant multidisciplinary meetings and the systematic assessment of the immunologic compatibility at the time of the organ offer for sensitized patients can safely and effectively broaden the donor pool.

### Combination of pretransplant desensitization therapies

There is a strong rationale to combine immunosuppressive/immunomodulatory agents to obtain synergistic effects. Complexity and redundant cellular functions of the alloimmune response following exposure to foreign HLA may explain the limited effect of isolated pharmacologic interventions. The combination of CoB and plasma cell depletion successfully resulted in a stable response to desensitization in preclinical models and humans. Depleting circulating antibodies could further augment this combination strategy. This theoretical framework allows for new agents that separately target plasma cells and memory B cell differentiation to be tested in combination. Synergistic combinations of therapies may outperform the conventional approach of administering add-on therapies for patients that have not responded to monotherapy.

### Individualization of desensitization therapies

Another critical point remains the identification of the appropriate therapy for each patient. Different modes of sensitization may result in heterogenous populations of plasma cells with distinct biological properties and memory B-cells with distinct activation requirements.^20^, ^34^ Attempts to identify the most likely responders to therapy are of particular interest to guide individualization of treatment. An in-depth evaluation of the recipient's immune state may contribute to revolutionizing the field. In a recent study, the identification of circulating memory B-cells subset phenotypes distinguished patients with successful serologic responses to CD38-targeting desensitization therapies from patients with poor or no responses.^35^ A comprehensive evaluation of successes and failures of desensitization should always be considered when reporting the biological and clinical effects of therapies.

### Consideration of the individual benefit of transplantation

Consideration of individual risks is crucial to tailor and optimize patient care. Immunologic risk should always be balanced with the consequences of not undergoing transplantation and with the possibility of a durable alternative to transplant (individual benefit from transplantation). When analyzing results from kidney desensitization therapy studies, the thoracic transplant community should keep in mind that heart and lung transplantations can be urgent life-saving procedures. Therefore, in selected cases and preferably in transplant centers with expertise in the management of highly sensitized patients, crossing the HLA barrier at the time of transplant, may be a viable option.

### Combining strategies: Pretransplant desensitization and perioperative pharmacologic antibody-risk mitigation

A complementary strategy to pretransplant desensitization therapies is the management of DSA at the time of transplant, either because the patient remains highly sensitized despite desensitization or because HTx is considered as an immediate life-saving procedure.

Transplantation across preformed DSA may occur with a risk of early AMR and graft loss. The landscape of our immunological understanding is evolving as multiple studies have reported different strategies efficiently to mitigate acute antibody-mediated allograft injuries. These relatively recent data should convince the transplant community that reasonably crossing the immunological barrier can be performed safely without compromising important outcomes.

According to the estimated immunological risk of transplantation, as determined using a multimodal assessment of DSA risk (MFI, dilution, C1q binding, positivity of flow or lymphocytotoxicity crossmatches), various strategies might be proposed. These strategies range from the standard mechanical removal of DSAs using perioperative plasmapheresis for moderate-risk DSA,^2^, ^50^ to more comprehensive strategies for higher-risk situations including complement blockade^36^, ^37^ or direct pharmacologic antibody depletion (Imlifidase).^51^

Eculizumab is a recombinant humanized monoclonal antibody that binds to the terminal complement protein C5, inhibiting the cleavage of C5 into C5a and C5b, thereby preventing the release of the inflammatory mediator C5a and the formation of the membrane attack complex. In an open-label, single-arm prospective trial of eculizumab for high-immunological-risk HTx, complement inhibition was well tolerated and associated with favorable outcomes. When compared with the standard of care (plasmapheresis-IVIg), eculizumab was associated with a significant decrease in the risk of biopsy-proven AMR.^36^, ^37^

Imlifidase is an IgG-degrading endopeptidase derived from *Streptococcus pyogenes* which cleaves human IgG into F(ab′)2 and Fc fragments, thus preventing complement activation and antibody-dependent cellular cytotoxicity. In 2 phase I/II studies, imlifidase reduced or eliminated DSA and permitted HLA-incompatible transplantation in 24 of 25 patients. However, an expected rebound in DSA shortly after transplantation was associated with frequent and early AMR, which was mostly responsive to treatment.^52^ The use of imlifidase has recently been reported in a lung transplant recipient with favorable short-term outcomes.^51^

We advocate for the development of shared protocols since all transplant centers do not have a high-immunological risk program and that most programs have a limited number of sensitized candidates.

### Post-transplant care

A close monitoring of sensitized patients is crucial during the early post-transplant course. The early detection and vigorous management of AMR, even at a subclinical state, and DSA rebound is particularly important to prevent severe antibody-induced allograft damage.

## Perspectives

Pretransplant desensitization is a constantly evolving field; 2 critical points should be kept in mind: (1) the rapid increase in the number of highly selective bioengineered monoclonal antibodies/cell-therapies offers new opportunities in the management of sensitized patients, and (2) a discussion regarding the end-points of interest when reporting new desensitization strategies is necessary to better reflect the entire transplant journey.

### Next-generation desensitization therapies

Although not yet evaluated in clinical practice, numerous emerging therapies may have theoretically a potential interest in pretransplant desensitization.

#### Bispecific T-cell engager

Teclistamab is a humanized bispecific antibody that binds B-cell maturation antigen (BCMA) on late-stage B cells and plasma cells, and CD3 on cytotoxic T lymphocytes. In phase I/II study, teclistamab resulted in a high rate of deep and durable response in patients with triple-class-exposed relapsed or refractory multiple myeloma.^53^ A phase 1/2, open-label study of REGN5459 or REGN5458 to desensitize kidney transplant candidates is ongoing.^54^

#### Chimeric antigen receptor-T cells

A chimeric antigen receptor is a synthetic receptor that is engineered to recognize specific antigens on the surface of cells of interest. This receptor is made up of an extracellular domain that binds to the target antigen, a transmembrane domain, and an intracellular domain that activates T-cell signaling pathways. Chimeric antigen receptor-T cell therapies have improved the prognosis of various hematological malignancies refractory to standard therapies.^55^ The safety, efficacy, and pharmacodynamics of CART-B-cell maturation antigen and huCART-19 are currently being assessed in highly sensitized kidney transplant candidates in phase I/II clinical trials.^56^

### Clinical end-points of interest

Selecting the appropriate end-points to evaluate the efficacy of pretransplant desensitization therapies is crucial for accurately assessing their impact on patient outcomes. Focusing only on pretransplant MFI, antibody titer, and/or cPRA does not account for the fact that desensitization can also profoundly alter immune cell phenotypes, independently of their effect on reducing HLA antibodies and cPRA. These effects may contribute to modifying post-transplant alloimmune responses and mitigating the risk of early AMR. Additionally, while the pretransplant desensitization literature has usually provided information on short-term outcomes, intermediate- and long-term outcomes are of particular interest: (1) the profound immunosuppression induced by the combination of pretransplantation desensitization, induction therapies, and post-transplant immunosuppressive regimen may have a major impact on post-transplant infections and malignancies, and (2) the mitigation of early acute antibody-mediated allograft injury may not avoid the development of chronic antibody-mediated injury.^57^ Overall, a combination of short-term and long-term end-points, including rejection rates, graft survival, and patient survival, offers the most comprehensive approach to understanding the true benefits and limitations of desensitization therapies.^49^

## Conclusion

Managing allosensitization requires a multifaceted approach. Current desensitization protocols, though helpful, are limited in efficacy and highlight the need for novel, tailored strategies. Emerging therapies show promise in improving desensitization outcomes and enhancing transplant success. A combination of pharmacologic interventions combining different desensitization therapies or associating pretransplant desensitization with perioperative antibody-risk mitigation could further optimize patient outcomes. Future research should focus on post-transplant outcomes, complications related to overimmunosuppression, and patient-specific approaches to refine desensitization practices in thoracic transplantation.

## Authors contribution

All authors contributed to the conceptualization, bibliography search, writing, drafting, and editing of the article.

## Disclosure statement

No financial disclosures.

## Data Availability

Data sharing not applicable—no new data generated.
